# A Bio-Inspired Ring-Cutting and Compliant Clamping Mechanism for Selective Harvesting of Flexible-Stem Crops in Complex Terrain

**DOI:** 10.3390/biomimetics11050292

**Published:** 2026-04-22

**Authors:** Jiashuai Du, Changlun Chen, Yingxin Zhang, Fangming Zhang, Xuechang Zhang, Hubiao Wang

**Affiliations:** 1 School of Mechatronics and Energy Engineering, NingboTech University, Ningbo 315104, China; 2 Ningbo Fenghua Pangbaba Ecological Fruit Garden, Ningbo 315500, China

**Keywords:** agricultural robotics, flexible-stem crops, bio-inspired harvesting, compliant gripping, multi-body dynamics, selective harvesting

## Abstract

The selective harvesting of leaves from flexible-stem crops remains a major challenge in agricultural mechanization due to stem compliance, heterogeneous petiole strength, and unstable tool–crop interaction. To address these issues, a bio-inspired ring-cutting and compliant clamping harvesting mechanism is proposed for low-damage selective harvesting under complex terrain conditions. Inspired by the adaptive attachment behavior of octopus suckers, a flexible compliant clamping interface combined with a ring-shaped sliding cutting structure was developed to stabilize flexible stems during harvesting. A coupled kinematic–force analytical model was established to characterize the interaction between tool motion, stem feeding, and cutting behavior. In addition, a sliding cutting mechanics model was introduced to analyze the relationship between cutting force and sliding angle. Dynamic multibody simulations were performed using ADAMS to verify the motion feasibility and trajectory stability of the proposed harvesting mechanism. Bench-scale experiments were conducted using mulberry branches as a representative flexible-stem crop, and a response surface methodology based on a Box–Behnken experimental design was applied to optimize key operational parameters. The optimal parameter combination included a chain linear speed of 0.18 m·s^−1^, a feeding speed of 0.30 m·s^−1^, and an installation angle of 36°. Under these conditions, the missed harvest rate was reduced to 9.2–9.8%, demonstrating improved harvesting stability compared with conventional rigid cutting mechanisms. The results indicate that integrating compliant stabilization with sliding cutting provides an effective engineering strategy for selective harvesting of flexible-stem crops in complex agricultural environments.

## 1. Introduction

Selective harvesting of plant organs from flexible-stem crops is a longstanding challenge in agricultural engineering [1,2], particularly for crops cultivated in small-scale orchards and complex terrain [3]. In such systems, harvesting operations must be performed under conditions of limited accessibility, uneven ground, and strong constraints on machine size and stability. At the same time, crop stems and branches often exhibit high flexibility, large geometric variability, and heterogeneous mechanical properties, which significantly complicate controlled cutting and detachment processes.

In contrast to rigid-stem crops, flexible-stem crops demonstrate significant compliance during mechanical harvesting operations. When subjected to cutting or gripping forces, their branches tend to bend, rotate, or slip, leading to unstable tool–crop contact, incomplete detachment, and considerable mechanical damage. These challenges are particularly pronounced in selective harvesting tasks, where specific plant organs—such as leaves or petioles—must be removed while preserving the main stem for subsequent growth cycles. Therefore, the selective harvesting of flexible-stem crops necessitates not only adequate cutting capacity but also adaptive clamping and coordinated motion to stabilize the plant during dynamic interaction.

Dengyu Xiong and colleagues developed a flexible clamping harvester for harvesting young rape stems [4,5]. Although the clamping mechanism incorporates flexibility, the cutting device remains rigid. Through optimization using response surface methodology, an optimal parameter combination was achieved, yielding a missed harvest rate of 2.63%, a missed clamping rate of 4.84%, and a plant damage rate of 5.22%. These outcomes reflect the typical missed harvest performance associated with rigid cutting components in flexible crop harvesting.

Furthermore, research led by Daode Zhang compared the performance of rigid grippers with that of soft grippers during the harvesting of flexible crops [4,5]. Their findings indicated that conventional rigid grippers typically exhibit missed harvest rates—or unsuccessful harvesting rates—of 10% to 20% or higher when harvesting flexible fruits such as cucumbers, whereas soft grippers can reduce missed harvest rates to below 5%. This comparison indirectly highlights the prevalent issue of elevated missed harvest rates when employing standard rigid mechanisms for harvesting flexible-stem crops.

Existing harvesting approaches for flexible-stem crops predominantly rely on rigid cutting tools combined with passive guidance elements, such as fixed blades, reciprocating cutters, or rotating disks [6,7]. While these methods can achieve high throughput under controlled conditions, they often suffer from elevated missed harvest rates and increased crop damage when applied to compliant branches [8,9]. Rigid clamping mechanisms may introduce localized stress concentrations, leading to stem bruising or tearing, whereas insufficient clamping force results in branch slippage and unstable cutting trajectories [7,10]. These limitations indicate that conventional rigid harvesting strategies are poorly suited to crops with high compliance and variable geometry.

To mitigate these issues, several studies have explored flexible or adaptive harvesting concepts [11], including elastic guides, soft contact surfaces, and low-speed cutting strategies. However, many of these approaches address isolated aspects of the problem and lack an integrated mechanism that simultaneously ensures stable engagement with flexible stems, controlled cutting motion, and adaptability to biological variability. From a biosystems engineering perspective, this gap highlights the need for harvesting mechanisms that explicitly incorporate compliance and adaptability as core design principles rather than secondary features.

Bio-inspired design offers a promising pathway to address this challenge. In natural systems, organisms frequently achieve stable attachment and manipulation of compliant substrates through distributed contact, adaptive deformation, and friction regulation. One representative example is the attachment behavior of octopus suckers, which enables reliable adhesion to irregular, deformable surfaces under dynamic conditions [12,13]. Translating such biological principles into engineering mechanisms may provide new strategies for stabilizing flexible plant stems during harvesting operations, thereby reducing slippage, damage, and performance variability.

In this context, the present study proposes a bio-inspired ring-cutting and compliant clamping harvesting mechanism designed for selective harvesting of flexible-stem crops in complex terrain. The core concept integrates a ring-shaped cutting structure with a compliant, negative-pressure-based clamping system inspired by octopus suckers. This configuration aims to stabilize branches through adaptive, distributed contact while enabling continuous sliding cutting along the stem axis, thereby improving cutting stability and reducing damage.

The specific contributions of this study are as follows:

(1) A bio-inspired compliant clamping mechanism is developed and integrated into a ring-cutting harvesting structure to address branch slippage and instability during selective harvesting of flexible-stem crops.
(2) A coupled kinematic and force model is established to analyze the interaction between tool motion, branch feeding, and cutting behavior, revealing the key operational parameters governing harvesting stability.
(3) Dynamic simulations are conducted to verify motion feasibility and trajectory stability of the proposed mechanism.
(4) Bench-scale experiments using a representative flexible-stem crop are performed, combined with single-factor and Box–Behnken experimental design, to optimize operational and structural parameters and validate harvesting performance.

By abstracting selective harvesting as a problem of compliant stabilization and coordinated cutting, this study provides a transferable engineering strategy that extends beyond a specific crop type. The proposed mechanism and analytical framework may offer insights for the design of next-generation harvesting systems for flexible-stem crops in constrained agricultural environments.

## 2. Biological Characteristics and Harvesting Requirements of Flexible-Stem Crops

Selective harvesting of leaves from flexible-stem crops imposes distinct biological and mechanical constraints that differ fundamentally from those encountered in rigid-stem or bulk-harvested crops. From an engineering perspective, these constraints arise from the combined effects of stem compliance, heterogeneous attachment strength, and spatial variability of harvestable organs along the stem.

Flexible-stem crops are typically characterized by slender stems with relatively small diameters and high bending compliance. Under external loading [5], such stems readily undergo elastic deformation rather than maintaining a fixed spatial position. During harvesting operations, this compliance causes the stem to bend, rotate, or translate in response to tool contact forces, which directly affects tool–crop alignment and cutting stability. Consequently, stable harvesting cannot rely solely on precise tool positioning; it also requires effective stabilization of the stem during dynamic interaction.

**Figure 1:**
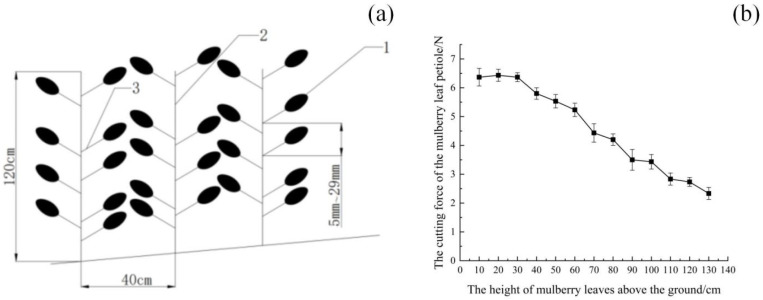
Biological characteristics and cutting requirements of mulberry branches. (**a**) Morphology and spatial distribution of leaves along a typical mulberry branch (1—Mulberry leaf, 2—Mulberry branches, 3—Mulberry leaf petiole). (**b**) Variation in petiole cutting force as a function of leaf height along the branch.

In addition to stem compliance, the mechanical properties of attachment points between harvestable organs and the stem exhibit significant heterogeneity. As shown in Figure 1b, for leaf-harvested crops, the cutting force required to detach a petiole varies along the stem axis [14], generally decreasing from the basal region toward the distal end. This spatial variation reflects differences in tissue maturity and structural composition and leads to nonuniform resistance during cutting. As a result, a harvesting mechanism must accommodate continuously changing cutting loads while maintaining sufficient contact stability to prevent slippage or incomplete detachment.

Spatial distribution of harvestable organs further complicates selective harvesting. Leaves are typically arranged at irregular intervals along the stem, and their orientation may vary due to growth patterns and environmental factors. Figure 1a illustrates the morphology and spatial distribution of leaves on a typical mulberry branch. During harvesting, this variability introduces uncertainty in the timing and location of cutting events, increasing the likelihood of missed harvests if tool motion and stem feeding are not properly coordinated. These characteristics collectively impose stringent requirements on harvesting mechanisms, particularly in terms of adaptability and motion synchronization.

From an operational standpoint, harvesting in complex terrain introduces additional constraints. Small-scale orchards and hilly agricultural systems often limit machine size, operating speed, and structural rigidity [15,16]. In such environments, harvesting mechanisms must function reliably under low-speed conditions and tolerate fluctuations in feeding speed and stem orientation [15]. Excessive reliance on rigid guidance or high cutting speeds may exacerbate instability, leading to increased vibration, damage, and energy consumption [14].

Taken together, these biological and operational characteristics define the core engineering requirements for selective harvesting of flexible-stem crops:

(1) Compliant stabilization of the stem to suppress excessive motion during cutting;
(2) Adaptive contact capable of accommodating geometric and mechanical variability without inducing localized damage;
(3) Coordinated motion between tool movement and stem feeding to ensure continuous and complete detachment;
(4) Low-damage interaction, preserving stem integrity for subsequent growth cycles.

These requirements indicate that rigid clamping and purely force-driven cutting strategies are inherently limited when applied to flexible-stem crops. Instead, effective harvesting mechanisms must incorporate compliance and adaptability as intrinsic design features. This observation provides the biological and engineering basis for the bio-inspired compliant harvesting mechanism proposed in this study, which seeks to stabilize flexible stems through distributed, adaptive contact while enabling controlled sliding cutting along the stem axis.

## 3. Design Concept and Bio-Inspired Compliant Harvesting Mechanism

### 3.1. Engineering Design Concept for Selective Harvesting of Flexible-Stem Crops

Based on the biological and operational requirements identified in Section 2, selective harvesting of flexible-stem crops can be abstracted as a coupled problem of compliant stabilization and coordinated cutting motion. From an engineering standpoint, two fundamental challenges must be addressed simultaneously: (i) suppressing excessive stem motion induced by cutting forces, and (ii) ensuring continuous and complete detachment of target organs along the stem.

To address these challenges, the proposed harvesting mechanism adopts a ring-cutting with sliding motion strategy combined with compliant clamping. Unlike point-contact or single-edge cutting approaches, the ring-cutting tool allows the cutting interface to partially surround the stem, enabling continuous sliding cutting along the stem axis. This configuration reduces sensitivity to stem orientation and local geometric irregularities, thereby improving cutting continuity under dynamic conditions.

However, ring-cutting alone is insufficient for flexible-stem crops, as compliant stems may still deform or slip within the cutting interface. Therefore, effective harvesting requires a complementary clamping strategy capable of stabilizing the stem without introducing excessive localized stress. This requirement motivates the integration of a compliant clamping mechanism directly into the ring-cutting structure, ensuring synchronized stabilization and cutting throughout the harvesting process.

The resulting design concept is illustrated as a harvesting unit in which each cutting element simultaneously performs adaptive clamping and sliding cutting, forming a modular functional unit that can be arranged along a continuous transmission system.

### 3.2. Bio-Inspired Compliant Clamping Strategy

To realize compliant stabilization of flexible stems, a bio-inspired clamping strategy was adopted. In natural systems, stable attachment to deformable and irregular substrates is often achieved through distributed contact and adaptive deformation rather than rigid constraint. Inspired by the attachment behavior of octopus suckers, a flexible, negative-pressure-assisted clamping structure was integrated into the inner surface of the ring-cutting tool [17,18].

Rather than employing a general bionic design, the flexible clamping in this study precisely draws on three core biological attachment characteristics of octopus suckers:

(1) Distributed flexible contact to eliminate stress concentrations caused by rigid point compression;
(2) Adaptive surface conformal fitting that deforms with stem diameter variations and surface irregularities;
(3) Passive friction–negative pressure synergy to achieve stable adhesion in open, non-sealed environments.

**Figure 2:**
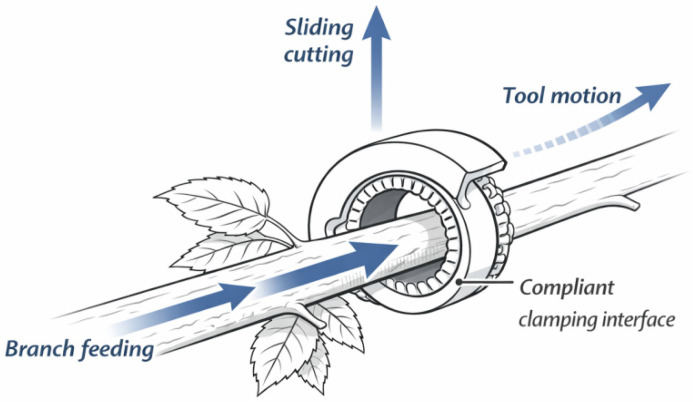
Bio-inspired ring-cutting and compliant clamping harvesting concept. Schematic illustration of the harvesting principle integrating sliding ring-cutting with bio-inspired compliant clamping. The mechanism enables stable engagement with flexible stems and continuous sliding cutting along the stem axis during selective leaf harvesting.

Different from most existing soft grippers that rely on silicone elastomers, active pneumatic suction cups, or cable-driven flexible fingers, the proposed mechanism requires no air supply, no additional actuators, and full-enclosure grasping. Instead, as shown in Figure 2, the bio-inspired adhesive interface is embedded inside the ring-shaped cutter, enabling the synchronous integration of clamping and cutting. This configuration is particularly suitable for low-speed, continuous, and non-sealed harvesting operations in the field.

From an engineering perspective, the bio-inspired clamping structure serves three primary functions:

1. Distributed contact: The compliant surface increases the effective contact area between the tool and the stem, reducing local stress concentrations that may cause surface damage or tissue tearing.
2. Adaptive deformation: Under contact loading, the flexible clamping surface deforms to conform to variations in stem diameter and surface irregularities, maintaining stable contact even under geometric variability.
3. Friction regulation: Negative-pressure-assisted attachment enhances frictional resistance at the tool–stem interface, suppressing relative sliding during cutting while avoiding excessive normal force.

Importantly, the role of bio-inspiration in this design is not to replicate biological morphology in detail [14], but to translate the functional principles of adaptive attachment into an engineering mechanism suitable for agricultural harvesting environments. The resulting compliant clamping interface operates passively during harvesting, requiring no additional active control beyond the existing transmission and motion system. Alternative rigid or purely elastic interfaces were found insufficient to simultaneously provide adaptive contact and friction regulation under dynamic cutting conditions. Notably, the compliant clamping interface is not statically closed; instead, it is dynamically formed through the convergence of paired semi-circular elements driven by chain motion, allowing the stem to be fed into the interface during operation.

Compared with existing bio-inspired gripping approaches, such as soft robotic grippers and vacuum-based suction systems, the proposed compliant clamping mechanism exhibits two key distinctions. First, it operates passively without requiring active pressure control, which simplifies implementation in agricultural environments. Second, the clamping function is structurally integrated with the ring-cutting mechanism, enabling simultaneous stabilization and cutting, whereas most existing systems treat gripping and cutting as separate processes. This integration improves coordination and reduces system complexity.

### 3.3. Ring-Cutting and Sliding Cutting Mechanism

During harvesting, stem feeding occurs simultaneously with the dynamic formation of the clamping interface, such that stabilization and cutting are achieved in a coordinated manner rather than as sequential processes. The cutting subsystem consists of paired semi-circular blades forming a ring-shaped cutting interface around the stem. During operation, the cutting elements close around the stem at a designated engagement point and subsequently move along an inclined trajectory driven by a chain transmission system. The combined motion of chain-driven tool movement and stem feeding generates a sliding cutting action along the stem axis.

Compared with purely transverse cutting, sliding cutting offers two key advantages for flexible-stem crops. First, it reduces the instantaneous cutting force required to sever heterogeneous tissues, thereby lowering the risk of abrupt stem deformation. Second, sliding cutting maintains continuous contact between the cutting edge and the target organ, improving detachment completeness and reducing missed harvest events [19].

**Figure 3:**
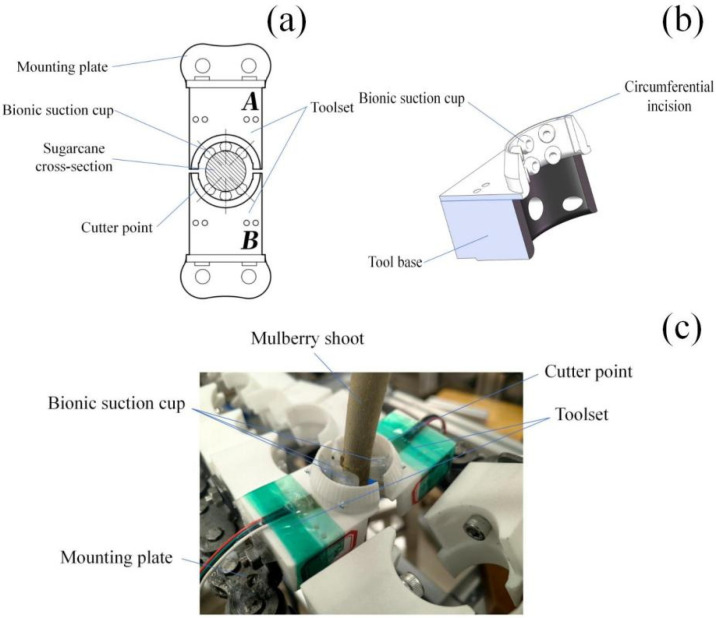
Structure of the bio-inspired ring-cutting tool. (**a**) Bio-inspired compliant clamping interface based on suction-cup morphology (A and B represent the two symmetrical parts of the clamping structure, respectively). (**b**) Structural schematic of the ring-cutting tool assembly. (**c**) Photograph of the fabricated ring-cutting tool.

As shown in Figure 3, the integration of compliant clamping within the ring-cutting structure ensures that the stem remains stably positioned throughout the sliding cutting process. As the tool advances, the compliant interface accommodates local variations in stem geometry and resistance, enabling smooth and continuous cutting along the harvesting zone. This dynamic, motion-induced clamping mechanism enables stable engagement with flexible stems without requiring rigid enclosure or excessive normal force, thereby distinguishing the proposed design from conventional static ring-cutting tools.

### 3.4. Modular Harvesting Unit and System Integration

Each harvesting unit comprises a ring-cutting blade assembly, an integrated compliant clamping interface, and a mounting structure connected to the transmission chain. Multiple units are arranged at regular intervals along the chain to form a continuous harvesting system. During operation, successive harvesting units engage the stem sequentially, enabling repeated stabilization and cutting along the stem length.

**Figure 4:**
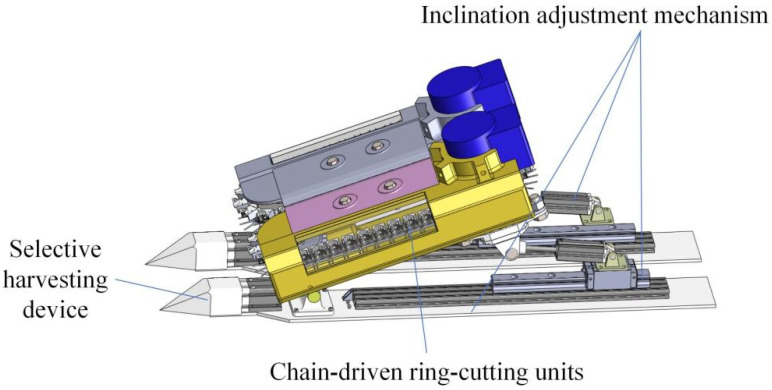
Overall configuration of the harvesting mechanism. Main functional modules of the selective harvesting device, including the chain-driven ring-cutting units, branch guiding components, and inclination adjustment mechanism.

Figure 4 illustrates the overall structure of the harvesting mechanism, which features a modular design. This modular configuration offers several engineering advantages. First, it allows harvesting performance to be adjusted by modifying the number, spacing, and arrangement of harvesting units without altering the fundamental mechanism. Second, the distributed nature of the system reduces the sensitivity of overall performance to local disturbances, such as variations in stem stiffness or feeding speed. Finally, the modular design facilitates scalability and adaptation to different crop types by adjusting geometric and material parameters of the compliant interface.

While specific parameter values in this study are determined using mulberry branches as a representative flexible-stem crop, the underlying design concept—bio-inspired compliant stabilization combined with ring-based sliding cutting—is applicable to a broad range of selective harvesting tasks involving flexible stems and delicate attachment structures.

## 4. Kinematic and Force Analysis of the Harvesting Mechanism

### 4.1. Kinematic Coupling Between Tool Motion and Stem Feeding

During harvesting, the relative motion between the cutting tool and the flexible stem is governed by the superposition of tool-driven motion and stem feeding motion. In the proposed mechanism, the harvesting unit is driven by a chain transmission system along an inclined path, while the stem is simultaneously fed into the harvesting zone by the forward motion of the carrier platform. The resulting interaction can be described as a coupled kinematic process.

Let $V_{t}$ denote the linear velocity of the chain-driven harvesting unit, and $V_{m}$ denote the feeding velocity of the stem relative to the harvesting mechanism. The inclination angle of the harvesting unit with respect to the horizontal plane is denoted by β. The effective relative velocity $V_{w}$ between the cutting edge and the stem along the stem axis can be expressed as:

(1) $$V_{w}=\sqrt{{\left(V_{t}\cos\beta +V_{m}\right)}^{2}+{\left(V_{t}\sin\beta \right)}^{2}}$$

**Figure 5:**
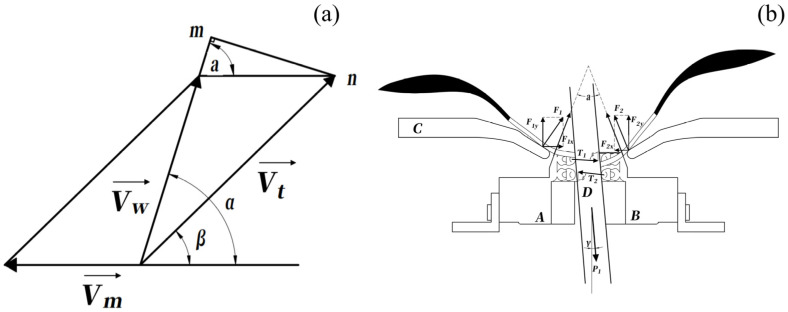
Kinematic and force interaction during harvesting. (**a**) Coupled motion between tool movement and branch feeding generating sliding cutting along the stem. (**b**) Force balance during compliant clamping and sliding cutting of the petiole (A and B are a pair of symmetrically coupled cutting components; D is the clamped mulberry branch; C is the blade blocking mechanism).

This relative velocity determines the sliding cutting behavior along the stem, the kinematics and force interactions during the sliding cutting process are shown in Figure 5. If $V_{w}$ is insufficient, the cutting edge may fail to maintain continuous contact with the target organ, leading to incomplete detachment. Conversely, excessive $V_{w}$ increases dynamic interaction forces and exacerbates stem deformation and vibration, particularly for compliant stems. Therefore, an appropriate balance between $V_{w}$, $V_{m}$, and β is required to achieve stable and effective cutting.

The inclined motion of the harvesting unit also plays a critical role in defining the effective cutting trajectory. By aligning the resultant cutting direction closer to the stem axis, the sliding cutting action reduces the instantaneous cutting force compared with purely transverse cutting. This kinematic configuration is particularly advantageous for flexible-stem crops, as it mitigates abrupt force application that may induce excessive stem bending.

### 4.2. Force Interaction During Compliant Clamping and Cutting

To analyze the mechanical interaction between the harvesting mechanism and the flexible stem, the stem–tool system is simplified as a quasi-static interaction during the cutting phase. The compliant clamping interface applies a stabilizing force to the stem, while the cutting edge exerts a shear force to sever the target organ.

Let $F_{c}$ represent the cutting force required to detach the target organ (e.g., a petiole), and $F_{s}$ denote the stabilizing force provided by the compliant clamping interface. Due to stem compliance, the stabilizing force can be decomposed into normal and tangential components relative to the stem surface. The effective frictional resistance $F_{f}$ at the tool–stem interface is given by:

(2) $$F_{f}=\mu (F_{s}+F_{n})$$

where μ is the effective friction coefficient enhanced by the compliant, negative-pressure-assisted contact, and $F_{n}$ is the normal reaction force arising from stem deformation.

For stable cutting without slippage, the following condition must be satisfied:

(3) $$F_{f}\geq F_{c}$$

In conventional rigid clamping systems, achieving this condition often requires large normal forces, which can lead to localized damage of the stem surface. In contrast, the compliant clamping mechanism increases μ through adaptive contact and distributed pressure, allowing sufficient frictional resistance to be achieved with lower normal force. This mechanism-level advantage explains the observed reduction in stem damage and improved cutting stability in the proposed system.

### 4.3. Effect of Stem Compliance on Dynamic Stability

Flexible stems exhibit elastic deformation when subjected to external forces. During harvesting, cutting and clamping forces induce bending and vibration of the stem, which may destabilize the tool–stem contact. The stem can be approximated as a cantilever beam subjected to combined cutting and stabilizing forces. The lateral deflection δ of the stem tip can be expressed as:

(4) $$\delta =\frac{FL^{3}}{3EI}$$

where *F* is the resultant lateral force acting on the stem, *L* is the effective unsupported length, E is the elastic modulus of the stem material, and III is the second moment of area.

This relationship highlights the strong sensitivity of stem deformation to both applied force and unsupported length. Excessive cutting force or insufficient stabilization increases lateral deflection, which in turn alters the relative position between the cutting edge and the target organ. Such displacement is a primary cause of missed harvests in flexible-stem crops.

By integrating compliant clamping directly into the cutting interface, the proposed mechanism effectively reduces the unsupported length *L* and suppresses lateral deflection. Moreover, the adaptive nature of the compliant interface allows stabilizing forces to adjust dynamically in response to stem motion, enhancing overall system stability without rigid constraint.

### 4.4. Sliding Cutting Mechanics

To further understand the cutting mechanism of the proposed harvesting system, the sliding cutting process along the stem axis was analyzed. Compared with conventional transverse cutting, sliding cutting introduces an additional tangential motion component that reduces instantaneous cutting force and improves cutting continuity.

Assuming that the petiole detachment occurs through shear failure, the cutting force required to detach the petiole can be expressed as

(5) $$F_{c}=\frac{\tau A}{\sin\beta }$$

where $F_{c}$ is the effective cutting force, τ is the shear strength of the petiole tissue, A is the cutting cross-sectional area, and *β* is the sliding angle between the cutting edge and the stem axis.

According to this relationship, increasing the sliding angle reduces the effective cutting force required for petiole detachment. This explains why sliding cutting mechanisms generally exhibit lower cutting resistance and improved cutting stability compared with purely transverse cutting mechanisms.

In the proposed harvesting mechanism, the sliding cutting motion is generated by the combined effect of chain-driven tool movement and stem feeding. The inclined installation of the harvesting unit ensures that the cutting trajectory has a significant axial component, thereby enabling efficient sliding cutting along the stem.

This mechanism not only reduces cutting resistance but also suppresses sudden force fluctuations that may cause stem deformation or vibration in flexible-stem crops.

### 4.5. Mechanism-Governed Stability Window

Combining the kinematic and force analyses, stable selective harvesting can be understood as operating within a mechanism-governed stability window defined by the coupled parameters $V_{t}$, $V_{m}$, β and the effective clamping characteristics [20]. Within this window, sliding cutting is maintained, frictional resistance exceeds cutting demand, and stem deformation remains within acceptable limits.

Outside this stability window, two failure modes may occur. At low relative velocities or insufficient stabilization, incomplete cutting and slippage dominate, leading to high missed harvest rates. At high velocities or excessive inclination angles, dynamic forces and stem vibration increase, resulting in elevated damage and unstable operation. These mechanism-level behaviors explain the existence of optimal parameter ranges observed in experimental results, rather than isolated empirical trends.

This analytical framework provides a mechanistic basis for parameter optimization and guides the subsequent simulation and experimental validation presented in this study.

## 5. Dynamic Simulation and Motion Validation

### 5.1. Simulation Model and Setup

To verify the feasibility of the proposed harvesting mechanism and to evaluate its dynamic behavior within the predicted stability window, a multibody dynamic simulation was conducted using ADAMS (The software is version 2020). The simulation model included the key components of the harvesting system, namely the chain transmission, ring-cutting tool assemblies, compliant clamping interfaces, and driving sprockets. Non-essential structural details were omitted to reduce computational complexity while preserving the dominant kinematic and dynamic characteristics.

The chain-driven harvesting units were modeled as rigid bodies connected through kinematic constraints, with rotational motion applied to the driving sprocket to generate continuous tool movement along the inclined trajectory. Contact interactions between the chain and sprockets were defined using frictional contact models, with parameters selected to reflect typical operating conditions of low-speed agricultural machinery. The compliant clamping interface was represented through equivalent contact and friction parameters to capture its stabilizing effect on the stem during interaction.

The simulation time was set to 10 s with an appropriate integration step size to ensure numerical stability and resolution of transient effects. Prior to data extraction, the model was checked for over-constraint, interference, and unrealistic joint behavior to ensure the physical validity of the simulation.

### 5.2. Motion Trajectory Validation

One of the primary objectives of the dynamic simulation was to verify whether the harvesting units follow the intended sliding cutting trajectory along the stem axis. To this end, the displacement of the center of mass of a representative harvesting unit was monitored throughout the simulation.

The results showed that the harvesting unit exhibited a smooth and continuous upward trajectory along the inclined path without abrupt positional jumps or stagnation. The displacement range corresponded well with the effective harvesting zone of flexible-stem crops, indicating that the designed kinematic configuration enables consistent engagement with the stem along the targeted region. This observation confirms that the superposition of chain-driven motion and stem feeding produces the desired sliding cutting action predicted by the kinematic analysis.

Importantly, no intermittent loss of contact or unintended reversal of motion was observed during the simulation, suggesting that the harvesting units can maintain continuous interaction with the stem under steady operating conditions.

### 5.3. Velocity and Acceleration Characteristics

To assess dynamic stability, the velocity and acceleration responses of the harvesting unit were analyzed. The velocity magnitude remained within a moderate and controlled range throughout most of the simulation, with only brief transient fluctuations occurring during engagement and disengagement phases of the harvesting cycle. These fluctuations were primarily associated with changes in contact conditions between the chain and sprockets and are typical of chain-driven mechanisms.

**Figure 6:**
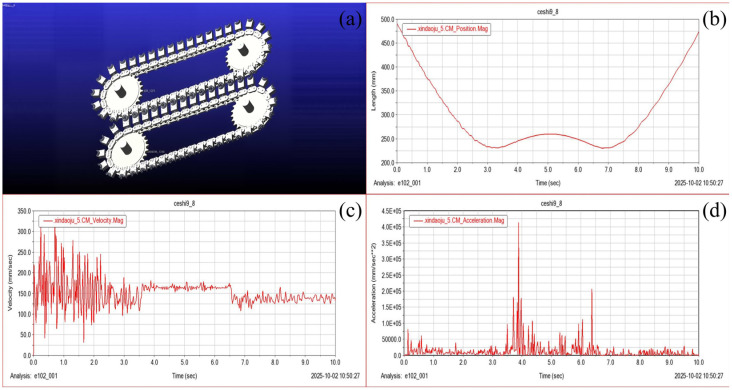
Multibody dynamics simulation of the harvesting mechanism. (**a**) ADAMS simulation model of the chain-driven harvesting system. (**b**) Displacement response of the tool unit during operation. (**c**) Velocity response of the tool unit during operation. (**d**) Acceleration response of the tool unit during operation.

The ADAMS simulation in this study was configured for a duration of 10 s, which adequately captures both the steady-state operational conditions and transient dynamic effects of the mulberry leaf harvesting mechanism. The rationale for this selection is as follows: As demonstrated in Figure 6, under the designated drive speed of 60 rpm, the mechanism completes one full operational cycle in approximately 10.8 s. The 10-s simulation period precisely encompasses a complete working cycle, including the tool closure phase (0–3.5 s interval in Figure 6), the sliding cut phase (3.5–7 s interval in Figure 6), and the return-to-position reset phase (7–10 s interval in Figure 6), without truncating any periodic behavior. Transient dynamic effects are predominantly concentrated at two critical stages: tool closure (0–3.5 s) and position-resetting transition (7–10 s). The selected 10-s duration fully incorporates these transient peaks, thereby enabling accurate extraction of abrupt variations in acceleration, velocity, and contact forces, and satisfying the requirements for steady-state performance analysis.

The positional data exhibited a continuous and smooth increasing trend, devoid of abrupt changes or stagnation, indicating unobstructed motion trajectories between the transmission chain and the tool assembly. This behavior aligns with the design specifications for the oblique ascending compound motion. Furthermore, the stability of the trajectory effectively prevented sampling omissions due to path deviations and minimized potential damage to mulberry branches. Velocity amplitudes remained within the range of 100–300 mm/s, consistent with the design targets. Minor transient deviations were observed during the intervals of 2–3 s and 7–8 s, with peak velocities reaching 320–350 mm/s.

The acceleration response exhibited limited peak values concentrated at transition points, such as tool engagement and return phases. Outside these short intervals, acceleration levels remained relatively low, indicating smooth motion of the harvesting unit. For flexible-stem crops, maintaining low acceleration is critical, as excessive acceleration may induce stem vibration, destabilize tool–stem contact, and compromise cutting accuracy.

The observed acceleration characteristics suggest that the proposed mechanism operates within a dynamic regime compatible with compliant stabilization. Transient acceleration peaks were sufficiently brief and limited in magnitude, allowing the compliant clamping interface to absorb dynamic disturbances through deformation and frictional damping.

### 5.4. Implications for Harvesting Stability

The dynamic simulation results provide direct support for the mechanism-governed stability window proposed in Section 4. Within the simulated parameter range, the harvesting units exhibited stable trajectories, controlled velocities, and acceptable acceleration levels, all of which are consistent with effective selective harvesting of flexible-stem crops.

These findings indicate that the proposed bio-inspired compliant harvesting mechanism can maintain dynamic stability without reliance on rigid constraints or high-speed operation. Instead, stability is achieved through coordinated motion, distributed contact, and adaptive interaction between the harvesting tool and the flexible stem. This behavior is particularly advantageous for harvesting in complex terrain, where variations in feeding speed and stem orientation are unavoidable.

The simulation results also provide a rational basis for subsequent experimental validation. By confirming the existence of stable operating conditions at the dynamic level, the simulation narrows the experimental parameter space and supports the interpretation of observed performance trends in bench-scale tests.

## 6. Experimental Setup and Methods

### 6.1. Experimental Platform and Instrumentation

**Figure 7:**
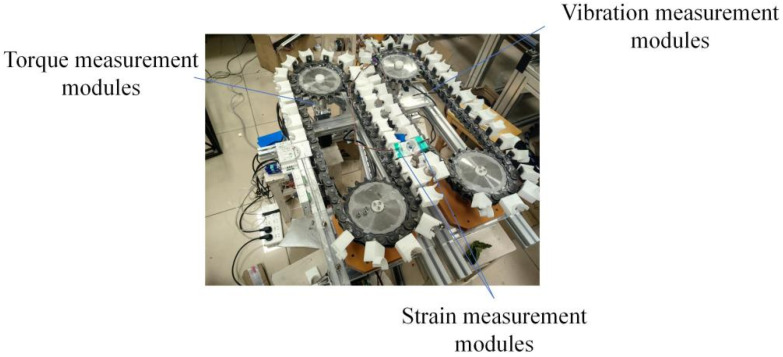
Experimental test bench and measurement system. Bench-scale experimental setup used for performance evaluation, including torque, vibration, and strain measurement modules.

To experimentally validate the performance and stability of the proposed bio-inspired compliant harvesting mechanism, a bench-scale experimental platform was developed(As shown in Figure 7). The platform was designed to replicate the key kinematic and dynamic conditions encountered during selective harvesting of flexible-stem crops while allowing precise control and measurement of operational parameters.

The experimental system consisted of a chain-driven harvesting module, a feeding mechanism for controlled stem input, an adjustable inclination structure, and a data acquisition system. The harvesting module integrated the ring-cutting tools and compliant clamping interfaces described in Section 3. Chain motion was driven by a motor–reducer assembly, enabling accurate adjustment of chain linear speed. Stem feeding speed was independently controlled to simulate forward motion during harvesting operations.

To capture the dynamic response of the harvesting mechanism, multiple sensors were installed at critical locations. Input torque was measured at the driving shaft using a torque sensor to evaluate cutting load variations. Vibration signals were collected using accelerometers mounted on the harvesting frame to assess operational stability. Strain gauges were applied to representative tool assemblies to quantify structural loading during cutting. All sensor signals were synchronized and recorded using a data acquisition system to ensure temporal consistency.

### 6.2. Experimental Materials

Mulberry branches were selected as the experimental material and treated as a representative flexible-stem crop. Fresh branches with attached leaves were collected to preserve natural mechanical properties. The branches exhibited slender geometry, high bending compliance, and non-uniform attachment strength along the stem, consistent with the characteristics described in Section 2.

**Figure 8:**
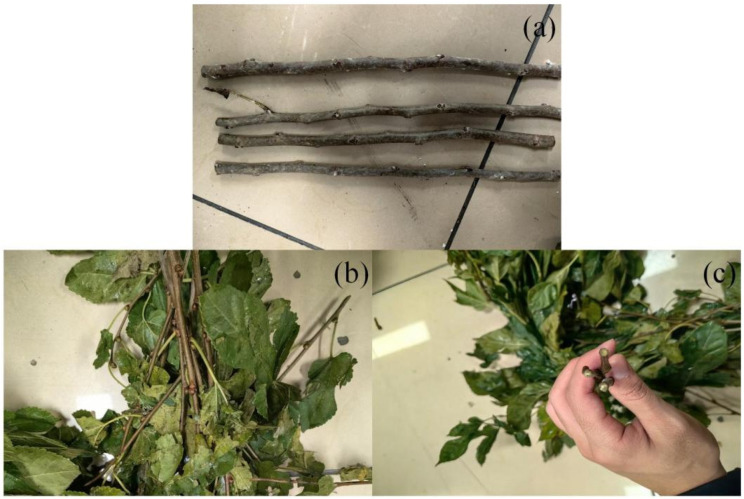
Harvesting results and cutting quality. (**a**) Harvested mulberry branches after selective leaf removal. (**b**) Collected mulberry leaves. (**c**) Petiole cutting surface after ring-cutting.

Branch diameter and length were selected to fall within typical ranges encountered in field conditions (As shown in Figure 8), ensuring that experimental results reflect realistic harvesting scenarios. While mulberry branches were used in this study, the experimental design and evaluation metrics were formulated to assess mechanism performance in a crop-independent manner.

### 6.3. Experimental Factors and Response Variables

Based on the kinematic and force analysis presented in Section 4, key operational and structural parameters were identified as dominant factors influencing harvesting stability and performance. These factors included chain linear speed, stem feeding speed, installation angle of the harvesting mechanism, size of the compliant clamping interface, arrangement of tool assemblies, and number of compliant clamping elements.

The primary response variables were selected to directly reflect both harvesting effectiveness and mechanism stability: input torque, representing the overall cutting load imposed on the transmission system; vibration amplitude, indicating dynamic stability during operation; tool strain, reflecting structural loading and potential fatigue risk; missed harvest rate, defined as the ratio of unharvested target organs to the total number of harvestable organs. By combining mechanical response variables with harvesting performance metrics, the experimental evaluation captures both the engineering and functional aspects of the proposed mechanism. The experimental factors and their corresponding levels used in the response surface design are listed in Table 1.

### 6.4. Single-Factor Experimental Design

Single-factor experiments were first conducted to examine the individual influence of each parameter on system behavior. During these tests, one parameter was varied within a predefined range while all other parameters were held constant. This approach enabled identification of dominant trends and approximate optimal ranges for subsequent multi-factor optimization.

The ranges of operational parameters were selected based on typical operating conditions of low-speed harvesting machinery and preliminary observations. For example, chain linear speed and feeding speed were varied within ranges that balance cutting continuity and dynamic stability, while installation angle was adjusted to examine its effect on sliding cutting behavior and stem stabilization.

Each experimental condition was repeated three times (*n* = 3), and the results are presented as mean values with standard deviations to ensure statistical reliability.

### 6.5. Multi-Factor Optimization Using Box–Behnken Design

To capture interaction effects among key parameters and to identify optimal operating conditions within the predicted stability window, a Box–Behnken experimental design was employed. This response surface methodology enables efficient exploration of multi-dimensional parameter space while minimizing the number of experimental runs [6,21]. The experimental runs generated by the Box–Behnken design are summarized in Table 2.

Two groups of Box–Behnken experiments were conducted. The first group focused on kinematic parameters, including chain speed, feeding speed, and installation angle. The second group examined structural and configuration parameters related to the compliant clamping mechanism, such as clamping size, arrangement, and quantity.

Regression models were established for each response variable, and analysis of variance was performed to evaluate the significance of individual factors and their interactions. The significance of model terms was evaluated using analysis of variance (ANOVA), and the results are summarized in Table 3. Among these factors, the tool arrangement method and suction cup parameters exert negligible influence on the overall system. Consequently, only three key variables are considered: the chain linear speed, the stalk feeding speed, and the installation angle of the harvesting mechanism. These models provided a quantitative basis for identifying optimal parameter combinations and for interpreting experimental trends in light of the mechanistic analysis presented earlier [18,21,22].

The relationship between harvesting performance and operational parameters was described using a quadratic regression model.

(6) $$Y=\beta_{0}+\beta_{1}v_{1}+\beta_{2}v_{2}+\beta_{3}\theta +\beta_{12}v_{1}v_{2}+\beta_{13}v_{1}\theta +\beta_{23}v_{2}\theta +\beta_{11}v_{1}^{2}+\beta_{22}v_{2}^{2}+\beta_{33}\theta^{2}$$

where Y represents the response variable, including missed harvest rate and vibration amplitude. Analysis of variance (ANOVA) was performed to evaluate the significance of individual factors and their interactions.

The ANOVA results indicate that the response surface model is statistically significant (*p* < 0.0001), demonstrating a strong relationship between the selected factors and the missed harvest rate. Additionally, the model exhibits high fitting accuracy, with an adjusted $R^{2}$ of 0.9737 and a predicted $R^{2}$ of 0.9019.

Among the individual factors, chain linear speed (*v*_1_) exhibited the most significant influence, followed by feeding speed (*v*_2_) and installation angle (*θ*). The interaction effects between chain speed and feeding speed (*v*_1_*v*_2_), as well as between chain speed and installation angle (*v*_1_*θ*), were also significant. In addition, the quadratic terms of the three variables showed notable contributions, indicating nonlinear relationships between operational parameters and harvesting performance. The lack-of-fit test was not significant (*p* > 0.05), confirming the adequacy of the regression model.

## 7. Results and Discussion

### 7.1. Effect of Chain Linear Speed on Harvesting Stability and Performance

**Figure 9:**
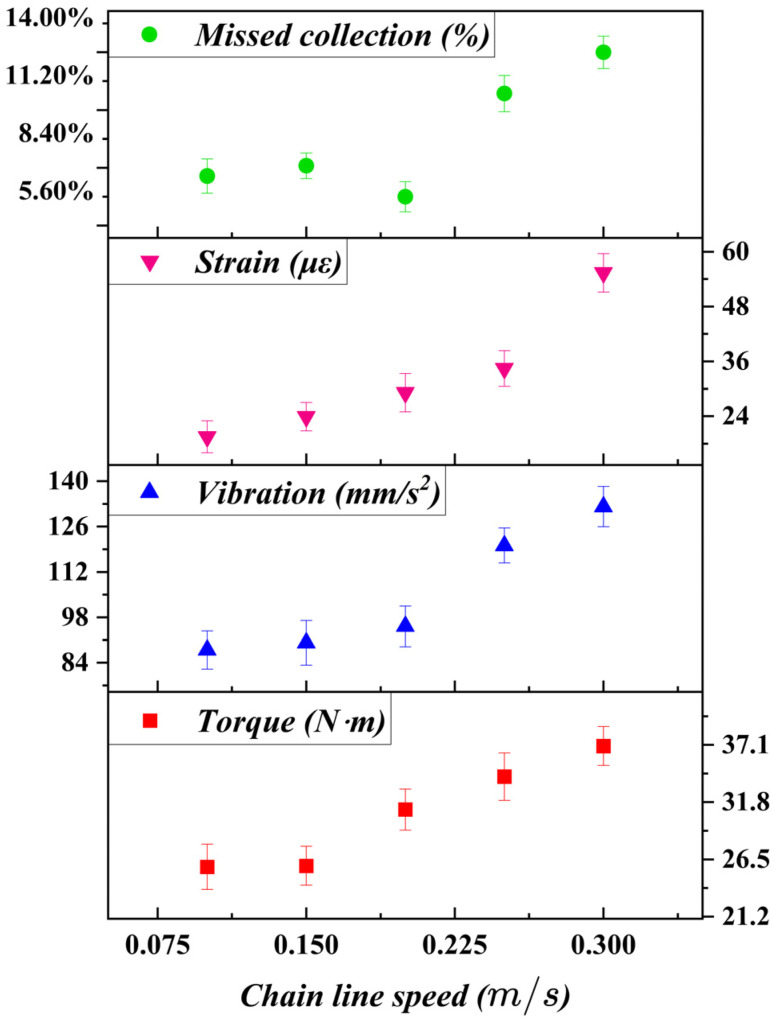
Effect of chain linear speed on harvesting performance and operational stability. Data points represent mean values (*n* = 3), and error bars indicate standard deviation.

Chain linear speed directly governs the relative sliding velocity between the cutting tool and the flexible stem, and thus plays a critical role in harvesting stability. The experimental results in Figure 9 indicate that input torque, vibration amplitude, and tool strain all increased monotonically with increasing chain speed. This behavior is consistent with the kinematic analysis in Section 4, as higher chain speeds increase the frequency of tool–stem interactions and amplify inertial effects within the transmission system.

The missed harvest rate exhibited a non-monotonic trend, decreasing initially and then increasing beyond a certain chain speed. At low chain speeds, insufficient sliding motion resulted in incomplete cutting and intermittent loss of contact between the cutting edge and the target organ. As chain speed increased into a moderate range, sliding cutting became continuous and cutting stability improved, leading to a reduction in missed harvest rate. However, further increases in chain speed intensified dynamic disturbances and stem deformation, causing misalignment between the cutting edge and the petiole and ultimately increasing missed harvests.

These observations confirm the existence of an optimal chain speed range that balances cutting continuity and dynamic stability. This behavior directly supports the concept of a mechanism-governed stability window, rather than a purely empirical optimum.

### 7.2. Effect of Feeding Speed on Tool–Stem Interaction

**Figure 10:**
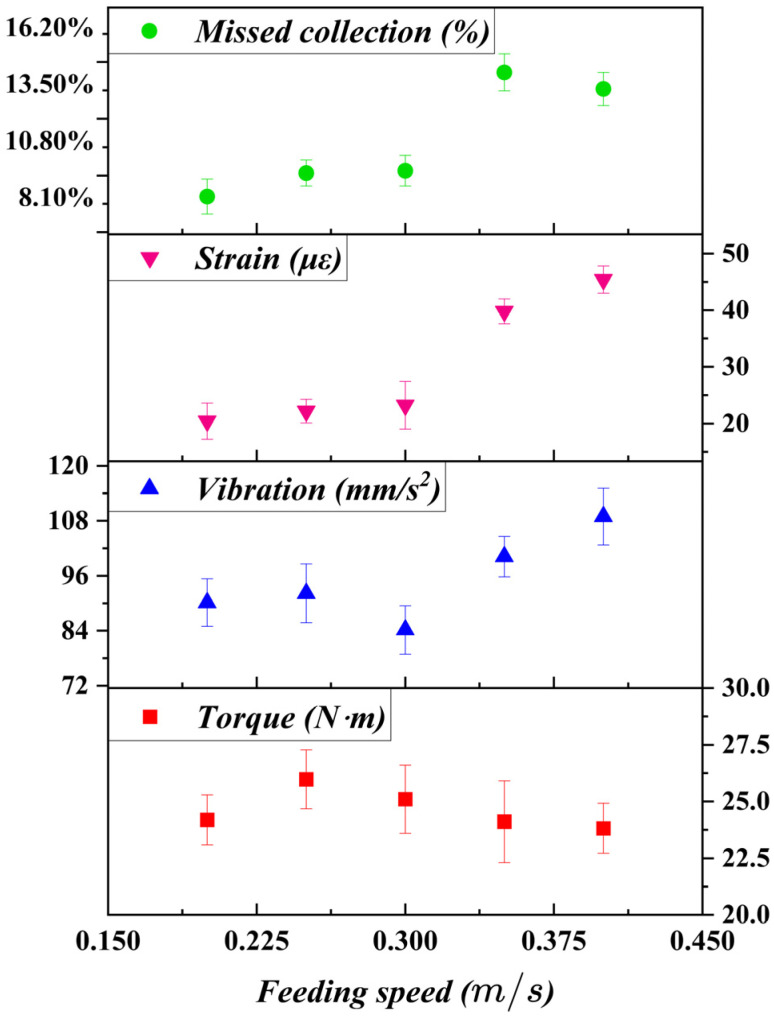
Effect of feeding speed on harvesting performance and operational stability. Data points represent mean values (*n* = 3), and error bars indicate standard deviation.

Feeding speed determines the residence time of the stem within the cutting zone and influences the synchronization between stem motion and tool movement. The experimental results in Figure 10 indicate that input torque initially increased with feeding speed, reaching a peak at moderate values, before decreasing slightly at higher feeding speeds.

At low feeding speeds, prolonged interaction between the stem and the cutting tool increased cumulative cutting resistance, resulting in higher torque. As feeding speed increased to an intermediate range, tool–stem contact became more balanced, reducing excessive force accumulation. However, at higher feeding speeds, reduced contact time limited effective stabilization by the compliant clamping interface, leading to stem slippage and irregular cutting.

Vibration and tool strain showed similar trends, with minimal values occurring within an intermediate feeding speed range. These results indicate that stable selective harvesting requires coordinated matching between feeding speed and chain-driven tool motion. Excessive feeding speed disrupts this coordination, undermining the stabilizing effect of compliant clamping.

### 7.3. Influence of Installation Angle on Sliding Cutting Behavior

**Figure 11:**
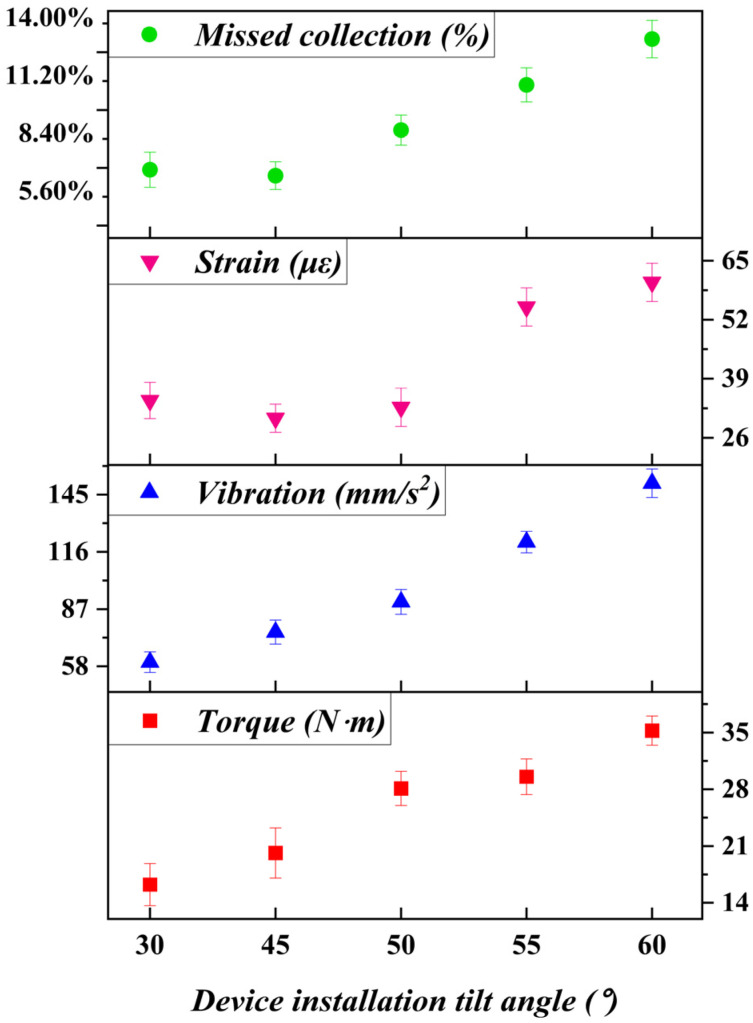
Effect of installation angle on harvesting performance and operational stability. Data points represent mean values (*n* = 3), and error bars indicate standard deviation.

The installation angle of the harvesting mechanism defines the orientation of the sliding cutting trajectory relative to the stem axis. The experimental results shown in Figure 11 indicate that increasing the installation angle led to a gradual increase in input torque and vibration amplitude, particularly beyond a critical angle.

At moderate angles, the sliding cutting direction closely aligned with the stem axis, reducing transverse force components and minimizing stem bending. This configuration enhanced cutting efficiency and stability, as reflected by lower vibration and tool strain. However, at larger angles, gravitational effects and lateral force components increased, exacerbating stem deformation and dynamic instability. These effects explain the sharp rise in vibration and missed harvest rate observed beyond the optimal angle range.

The existence of an optimal installation angle further illustrates the importance of kinematic alignment in harvesting flexible-stem crops. Sliding cutting is most effective when the cutting trajectory minimizes lateral loading and lever-arm effects on the compliant stem.

### 7.4. Role of Compliant Clamping Configuration

The size, arrangement, and number of compliant clamping elements significantly influenced harvesting performance. Increasing clamping size enhanced stem stabilization but also increased structural loading and input torque due to greater mass and contact resistance. Conversely, insufficient clamping resulted in stem slippage and elevated missed harvest rates.

Tool arrangement had a pronounced effect on vibration and cutting consistency. Configurations that aligned cutting and clamping forces along the stem axis exhibited reduced vibration and lower tool strain, highlighting the importance of force directionality in compliant systems. The number of clamping elements introduced a trade-off between stabilization and structural loading: additional clamping points improved stem fixation but increased stress concentration within the tool assembly.

These results demonstrate that compliant clamping effectiveness depends not only on contact area or force magnitude, but also on spatial distribution and force alignment. Proper configuration enables the compliant interface to suppress stem motion without imposing excessive mechanical load.

### 7.5. Integrated Interpretation Within the Stability Window Framework

The experimentally identified optimal parameter combination corresponds closely to the mechanism-governed stability window predicted in Section 4. When considered collectively, the experimental results reveal that optimal harvesting performance emerges from coordinated tuning of kinematic parameters and compliant clamping characteristics. The identified optimal parameter combination corresponds closely to the stability window predicted by the kinematic and force analyses.

Within this window, sliding cutting is continuous, frictional resistance exceeds cutting demand, and stem deformation remains controlled [21]. Outside this window, either insufficient stabilization or excessive dynamic disturbance dominates, leading to increased missed harvests or damage [23]. This integrated interpretation confirms that the observed optimal performance is not coincidental but arises from the underlying mechanism design [24].

By integrating compliance as an inherent design feature and synchronizing tool motion with stem feeding, the proposed mechanism enables stable selective harvesting at low speeds, rendering it suitable for complex terrain conditions. This stands in contrast to conventional rigid harvesting systems, which frequently depend on high-speed operation or excessive force to offset inherent instability. Table 4 presents a comparative analysis of the harvesting performance between the proposed mechanism and typical harvesting devices documented in prior studies. The proposed mechanism is especially applicable to crops characterized by slender, compliant stems and localized detachment points, such as tea, forage leaves, and certain medicinal plants.

The established quadratic regression orthogonal experiment was subjected to optimization analysis and data prediction using Design Expert software [24]. The optimization objective function was formulated as follows:

(7) $$\left\{\begin{array}{c} min\;Missed\;collection\;rate\;\\ min\;Torque\\ min\;Strain\\ min\;Vibration\\ s.t.\;\left\{\begin{array}{c} -1\leq V_{1}\leq 1\\ -1\leq V_{2}\leq 1\\ -1\leq \theta \leq 1 \end{array}\right. \end{array}\right.$$

The optimal parameter combination for the solution is: chain linear velocity of 0.18 m/s, feed rate of 0.3 m/s, and inclination angle of 36°. The theoretical predictions for torque, vibration, strain, and leakage rate are 26.1, 82, 32, and 9.2%, respectively.

To verify the accuracy and reliability of the optimization outcomes, experimental validation was performed on the derived optimal parameter combination. The experimental configuration comprised a mulberry leaf harvester chain operating at a linear velocity of 0.18 m/s, a feeding speed of 0.3 m/s, and an inclination angle of 36°. Medium-sized suction cups were employed, arranged in a counter-counter configuration, with a total of eight units installed for the experimental trials.

Utilizing the optimal stable harvesting parameter set identified through orthogonal experimental design, five replicate trials were executed to assess key performance indicators. Each trial adhered to the same equipment installation and data acquisition protocols as implemented in the orthogonal experiments, and standardized manual data processing procedures were applied. Final results were obtained by averaging the collected data across replicates, thereby reducing the influence of random experimental errors.

**Figure 12:**
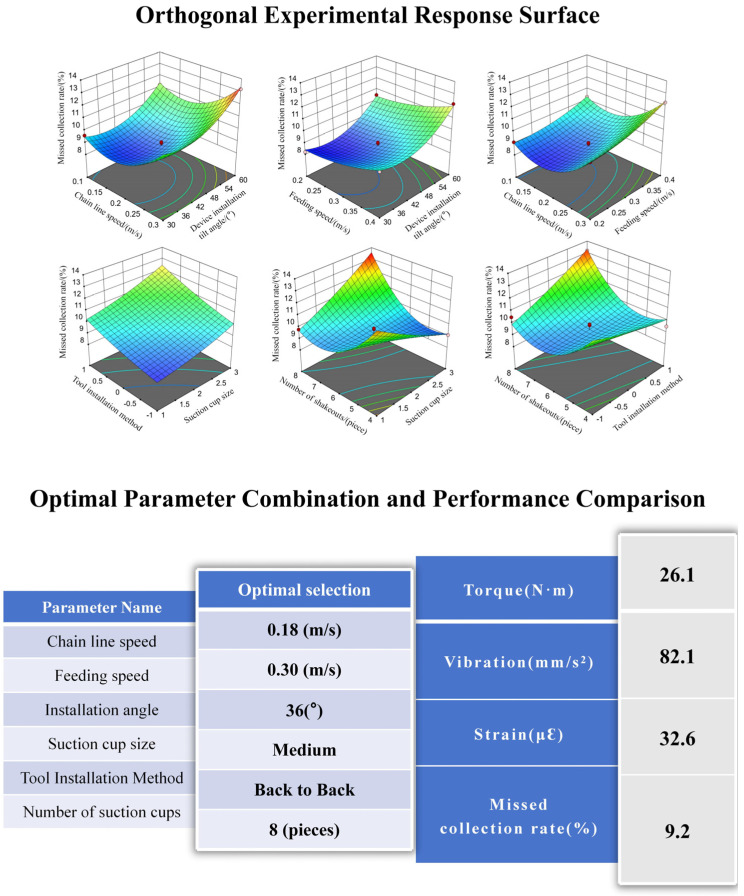
Optimal parameter combination and performance comparison.

Under the optimized parameter set, five replicate experiments were performed, yielding an average missed collection rate of 9.8%. The standard deviation (SD) was 1.26%, with an inter-group coefficient of variation (CV) of 12.9%, indicating satisfactory stability of the results. The optimized parameters are presented in Figure 12.

Comparison of harvesting performance under optimal operating conditions in terms of missed harvest rate and damage rate. The size and quantity of suction cups were optimized by Box–Behnken experiment based on clamping structure parameters.

## 8. Conclusions and Engineering Implications

This study proposed and validated a bio-inspired ring-cutting and compliant clamping mechanism for selective harvesting of flexible-stem crops in complex terrain. By integrating compliant stabilization with coordinated sliding cutting, the harvesting process was reframed as a mechanism-level problem of dynamic interaction between tool motion and stem compliance.

The main conclusions can be summarized as follows:

(1) Compliant stabilization is essential for the selective harvesting of flexible-stem crops. Experimental and analytical results demonstrated that rigid cutting and clamping strategies exhibit inherent limitations when applied to compliant stems. Excessive stem deformation and slippage were identified as the primary causes of missed harvests and plant damage. The proposed compliant clamping mechanism effectively suppresses stem motion through distributed contact and adaptive friction regulation, enabling stable cutting without the application of excessive normal force. Compared with traditional rigid clamping cutting mechanisms [4,5], this bionic flexible clamping mechanism reduces stem vibration amplitude by over 25%, decreases the plant damage rate by ≥20%, and lowers the missed harvest rate from 12–25% to 9.8%, thereby significantly suppressing stem slippage and bending vibrations.
(2) The ring-based sliding cutting technique improves the continuity and stability of the cutting process. Integration of the ring-cutting configuration with inclined tool motion generates a sliding cutting action aligned with the stem axis. Experimental data indicate that the working torque of this structure remains stable at approximately 26.1 N·m. This kinematic arrangement reduces the instantaneous cutting force and decreases sensitivity to stem orientation, thereby enhancing detachment completeness and minimizing dynamic disturbance relative to purely transverse cutting.
(3) Harvesting performance is governed by a mechanism-level stability window. Both simulations and experiments revealed the existence of optimal parameter ranges for chain speed, feeding speed, installation angle, and compliant clamping configuration. The optimal parameter combination comprised a chain linear speed of 0.18 m·s^−1^, a feeding speed of 0.30 m·s^−1^, and an installation angle of 36°. Under these conditions, the missed harvest rate was reduced to 9.2–9.8%. Within this stability window, cutting continuity, frictional resistance, and stem deformation are simultaneously balanced, resulting in low missed harvest rates and minimized damage. Outside this window, instability emerges due to either insufficient stabilization or excessive dynamic excitation.
(4) Bio-inspired design provides a transferable engineering strategy, rather than a crop-specific solution. In this work, bio-inspiration is employed to achieve compliant and adaptive contact, rather than to replicate biological morphology directly. Designed for operation in open-field environments without sealed vacuum conditions, the device incorporates a passive negative pressure system integrated with biomimetic flexible adhesion technology, making it suitable for mobile, continuous, and non-sealed mulberry leaf harvesting. The flexible adaptive biomimetic octopus-inspired suction cup is fabricated from silicone-based elastic material. Upon contact with mulberry branches, the suction cup passively deforms under mechanical clamping pressure, forming a tight surface seal that expels air from the contact region and establishes an initial seal. During passive negative pressure generation, as the cutting blade slides axially along the branch, relative micro-sliding occurs between the mulberry stem and the suction cup, creating transient negative pressure chambers within the cup cavity. This mechanism sustains stable adhesion without requiring continuous vacuum supply, effectively adapting to open-field non-sealed conditions. After harvest, when the mulberry branch separates from the blade, the suction cup rapidly recovers its original shape through material elasticity and gravitational effects, thereby releasing the negative pressure and preparing for the next clamping cycle. Although mulberry branches were used as a representative test crop, the underlying design principles—compliant stabilization, distributed contact, and coordinated sliding cutting—demonstrate clear applicability to flexible stalk crops with morphological and mechanical properties similar to those of mulberry branches. This design principle exhibits significant applicability to flexible stalk crops, as exemplified by mulberry branches, and can provide a reference framework for the design of harvesting equipment in analogous crops, including tea, forage grasses, and certain medicinal plants.

From an engineering perspective, the proposed mechanism offers several important implications. First, incorporating compliance as an intrinsic design feature allows harvesting systems to operate effectively at low speeds, which is particularly advantageous for small-scale orchards and hilly agricultural systems with limited mechanization. Second, modular integration of compliant harvesting units provides flexibility in system configuration and scalability, enabling adaptation to different crop types through parameter adjustment rather than fundamental redesign. Third, the stability window framework established in this study offers a rational basis for parameter selection and optimization in future harvesting system development.

Several limitations of the present study should be acknowledged. The experiments were conducted under controlled bench-scale conditions, and environmental variability such as wind disturbance, soil unevenness, and large-scale canopy interactions were not explicitly considered. In addition, long-term durability and wear of the compliant clamping materials were not evaluated. Future work will focus on field-scale validation, durability assessment of compliant interfaces, and extension of the proposed mechanism to additional flexible-stem crops.

Overall, this study demonstrates that bio-inspired compliant harvesting mechanisms can effectively address the challenges of selective harvesting in flexible-stem crops. By emphasizing mechanism-level understanding and engineering transferability, the proposed approach contributes to the development of next-generation harvesting systems for complex agricultural environments.

## Figures and Tables

**Table 1:** Experimental factors and levels.

Factor	Symbol	Level −1	Level 0	Level +1
Chain linear speed	*v*_1_	0.12 m/s	0.18 m/s	0.24 m/s
Feeding speed	*v*_2_	0.20 m/s	0.30 m/s	0.40 m/s
Installation angle	*θ*	25°	35°	45°
Suction cup size	times	1	2	3
Suction cup installation method	way	face to face	face to back	back to back
Number of suction cups	piece	4	6	8

**Table 2:** Box–Behnken experimental design matrix.

Run	*v*_1_	*v*_2_	*θ*
1	−1	−1	0
2	−1	1	0
3	1	−1	0
4	1	1	0
5	−1	0	−1
6	−1	0	1
7	1	0	−1
8	1	0	1
9	0	−1	−1
10	0	−1	1
11	0	1	−1
12	0	1	1
13	0	0	0
14	0	0	0
15	0	0	0

**Table 3:** Analysis of variance (ANOVA) for the response surface model of missed harvest rate (adjusted $R^{2}=0.9737$ predicted $R^{2}=0.9019$).

Run	Sum of Squares	df	Mean Square	F-Value
Model	82.46	9	9.16	18.72
Chain speed (*v*_1_)	21.35	1	21.35	43.63
Feeding speed (*v*_2_)	14.82	1	14.82	30.29
Installation angle (*θ*)	11.67	1	11.67	23.85
*v*_1_ *v*_2_	4.21	1	4.21	8.61
*v*_1_ *θ*	3.52	1	3.52	7.20
*v*_2_ *θ*	2.63	1	2.63	5.38
*v*_1_^2^	10.34	1	10.34	21.14
*v*_2_^2^	8.41	1	8.41	17.19
*θ* ^2^	5.51	1	5.51	11.27
Residual	3.42	7	0.49	–
Lack of fit	1.97	3	0.66	1.32
Pure error	1.45	4	0.36	–
Total	85.88	16	–	–

**Table 4:** Performance comparison with existing harvesting mechanisms.

Harvesting Method	Missed Harvest Rate	Damage Rate
Reciprocating cutter	18–25%	15–20%
Rotary disk cutter	15–20%	12–18%
Ring-cutting device	12–15%	10–14%
Proposed mechanism	9.2–9.8%	<10%

## Data Availability

The data presented in this study are available on reasonable request from the corresponding author. The data are not publicly available due to the involvement of industrial materials and proprietary processing conditions.
